# Multicolor labeling of airway neurons and analysis of parasympathetic heterogeneity

**DOI:** 10.1038/s41598-022-08655-6

**Published:** 2022-03-23

**Authors:** Alexandra B. Pincus, Samuel J. Huang, Katie M. Lebold, Ubaldo De La Torre, Becky J. Proskocil, Matthew G. Drake, Hiroyuki Nakai, Allison D. Fryer, David B. Jacoby

**Affiliations:** 1 Oregon Health & Science University, 3181 SW Sam Jackson Park Road, BRB 440, Portland, OR 97239 USA; 2 Department of Emergency Medicine, Stanford University School of Medicine, 900 Welch Rd, Palo Alto, CA 94304 USA

**Keywords:** Autonomic nervous system, Viral tracing, Respiration

## Abstract

We report subpopulations of airway parasympathetic neurons expressing substance P, neuronal nitric oxide synthase, and tyrosine hydroxylase, highlighting unexplored heterogeneity in this population. These neurotransmitter-specific subpopulations did not form intraganglionic interneurons, but rather, extended outside the ganglia, into the airways, to distant innervation targets. Our experiments demonstrate the utility of multicolor labeling to characterize airway innervation, allowing us to confirm the extensive heterogeneity of postganglionic parasympathetic neurons. These methods will facilitate future investigations of neurophysiology and neural contributions to airway disease.

## Introduction

Airway parasympathetic neurons are primarily cholinergic, releasing acetylcholine onto muscarinic receptors on airway smooth muscle to elicit bronchoconstriction and mucus secretion. However, immunostaining in human and ferret airways has also identified subsets of airway neurons that express substance P (SP)^1–3^, neuronal nitric oxide synthase (nNOS)^2,4^, and tyrosine hydroxylase (TH)^5^. The functions and innervation targets of these neuronal subpopulations remain unknown. Intriguingly, the prevalence of these unique neuronal subpopulations is increased in disease states, including increases in substance P-expressing neurons in ozone-mediated airway hyperreactivity^6^ and increases in tyrosine hydroxylase-expressing neurons post-lung transplant^5^. Understanding the connectivity of these subpopulations will provide new insights into their function and role in airway pathophysiology.

Multicolor labeling of nerves, such as in Brainbow mice^7^, allows distinct nerve processes in close proximity to be distinguished and traced to their target destinations. This technique generates fluorescent labeling over a wide spectrum of colors in Thy1 + neurons by using random combinations of three fluorophores. Notably, the original Brainbow technique cannot be applied to the study of airway nerves because it utilizes a Thy1 promoter to drive fluorophore expression, and the Thy1 promoter is not expressed in postganglionic parasympathetic nerves^8^. Thus, we employed a combinatorial multicolor viral vector labeling strategy, as has been employed in other organs^9^. In this approach, mice are simultaneously injected with three adeno-associated virus (AAV) vectors, each of which expresses a different fluorophore. Transduction produces a stochastic distribution of vector genome copies encoding fluorophores within each target cell and results in a wide variety of colors across neurons that can be distinguished and traced. Here, we first established the tropism of AAV9 vectors for parasympathetic nerves in the airways. We subsequently used immunohistochemistry to identify the cell bodies of SP-, nNOS-, and TH-expressing neurons within murine parasympathetic ganglia and applied AAV9-mediated combinatorial multicolor viral vector nerve labeling to trace individual neuronal processes and determine whether these cells represented intra-ganglionic interneurons or projected to more distant targets.

## Materials and methods

### Animals

Experiments used 8-week-old C57BL/6 mice or offspring from a cross between CHAT-Cre (#028861) and CH2-EYFP (#024109) mice from Jackson Labs. All mice were housed communally with unlimited access to food and water and 12 h light/dark cycle. Male and female mice were used for quantification of neurons expressing SP, nNOS, and TH, male mice were used for viral vector and tracing studies.

### Plasmids

**Figure 1:**
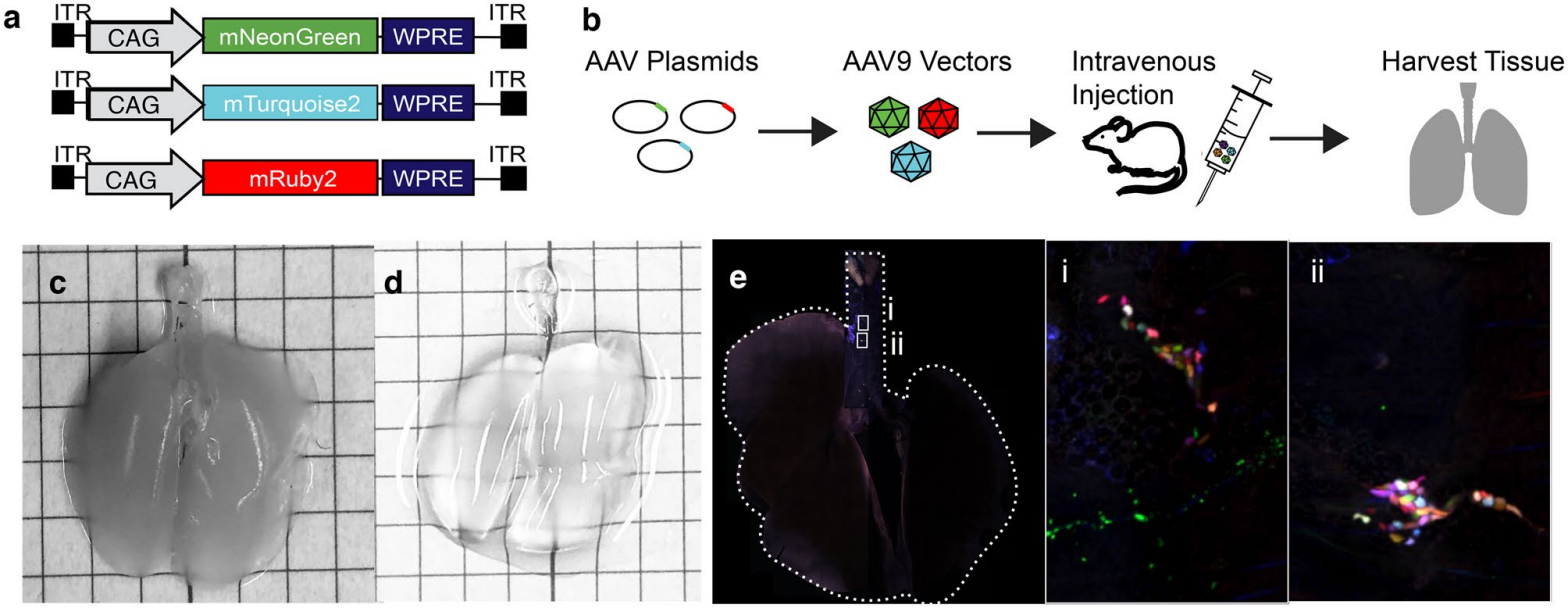
Multicolor nerve labeling approach and identification of parasympathetic subpopulations. (**a**,**b**) Schematic of multicolor AAV vector approach and AAV vector genome plasmids. Mouse airways before (**c**) and after (**d**) passive clearing in Ce3D. (**e**) Whole mouse trachea and lungs showing multicolor parasympathetic ganglia.

AAV vector genome plasmids harboring CAG-driven mNeonGreen, mRuby2, and mTurquoise2 transgene cassettes were purchased from Addgene. pAAV-CAG-mNeonGreen was a gift from Pantelis Tsoulfas (RRID:Addgene_124101). pAAV-CAG-mTurquoise2 (RRID:Addgene_99122) and pAAV-CAG-mRuby2 (RRID:Addgene_99123) were a gift from Viviana Gradinaru (Fig. 1a).

### Cell culture conditions and experiments

HEK293 cells were maintained in Dulbecco’s Modified Eagle’s Medium (Lonza) supplemented with 10% fetal bovine serum (Sigma-Aldrich), 1% L-glutamine (Lonza), and 1% penicillin–streptomycin (Lonza). mNeonGreen, mRuby2, and mTurquoise2 plasmids were independently transfected into HEK293 cells using Lipofectamine 2000 (Thermo Fisher) with 400 ng plasmid per well on an 8-well slide. Cells were imaged using confocal microscopy after 48 h. Microscope settings were optimized to limit bleed-through (Supplementary Fig. 1).

### AAV9 vector production, purification, titration, and administration

We produced AAV9 vectors in HEK293 cells (AAV-293, Agilent) using an adenovirus-free triple transfection method followed by polyethylene glycol 8000 precipitation and purification by two rounds of cesium chloride ultracentrifugation^10^. The final vector excipient was 5% sorbitol in PBS supplemented with 0.001% Pluronic-F68. Vectors were titered by radioactive dot blot assay^11^ and an equimolar mixture of mNeonGreen, mRuby2, and mTurquoise2 AAV9 vectors was administered intravenously via lateral tail vein without sedation at a total dose of 3 × 10^11^ to 3 × 10^12^ vg per mouse (Fig. 1b). For initial tests of viral dosage, AAV9 vectors expressing GFP were administered at doses of 5 × 10^10^ to 5 × 10^12^. Animals were euthanized 21 days after viral injection.

### Tissue immunohistochemistry, optical clearing and imaging of airway nerves

Mice were euthanized with pentobarbital (150 mg/kg i.p.) and perfused with PBS. Tracheas were excised and stored at 4 °C in Zamboni fixative (Newcomer Supply) overnight. For investigation of vector uptake into other neural types, DRGs, vagal ganglia, and superior cervical ganglia were also excited, and tissues were processed in the same manner. To image fluorescent nerves, tissues were washed with TBS and passively cleared in N-methylacetamide/Histodenz (Ce3D)^12^ overnight (Fig. 1c,d). For immunohistochemistry experiments, tracheas were blocked overnight with 4% goat serum, 1% Triton X-100, and 5% powdered milk, then incubated for 4 h at 4 °C with antibodies against PGP9.5 (rabbit anti-human PGP9.5, 1:500; Millipore AB1761-I), substance P (rat anti-SP, 1:500; BD Pharmigen 556312), neuronal nitric oxide synthase (rabbit anti-nNOS, 1:100; Cell Signal C7D7), or tyrosine hydroxylase (rabbit anti-TH, 1:500, Pel Freez P40101). Tracheas were washed and incubated overnight with secondary antibodies (Alexa goat anti-rat 647, 1:1,000; Alexa goat anti-rabbit 647, 1:1,000; Alexa goat anti-chicken 488, 1:1,000; Invitrogen) before clearing overnight in Ce3D. Tissues were mounted in Ce3D on slides in silicon wells (Grace Labs) and imaged using an LSM 900 confocal microscope with four separate lasers and acquisition tracks: 405 nm (detection wavelengths 467–503), 488 nm (516–526), 561 nm (576–619) and 640 nm (645–700). Images were acquired as z-stacks, with single plane and maximum intensity projections used for visualization. Imaris and Imaris Stitcher software were used for 3D visualization.

### nTracer analysis of parasympathetic nerve morphology

Tracing studies were performed on 8-bit images using FIJI software with the nTracer plugin^13^. Cell bodies for SP, nNOS, and TH neurons were identified with immunofluorescent labeling. Traces were created around the cell bodies of neurons and followed axons through each image. User inputs for points along the axon were verified through three-channel color intensity analysis in nTracer. Final traces represent a path of pixels with matching intensity in each color channel between user-identified points.

### Statistical analysis

Fluorescence intensity data for multicolor cell bodies was not normally distributed (D’Agostino and Pearson test), and were analyzed using a Kruskal–Wallis test with Dunn’s post-hoc using GraphPad Prism software. The study was carried out in compliance with ARRIVE guidelines^14^.

### Regulatory

All methods were approved by the Oregon Health & Science University’s Institutional Animal Care and Use Committee and Institutional Biosafety Committee and were performed in accordance with the relevant guidelines and regulations.

## Results

### Airway postganglionic parasympathetic neurons express substance P, neuronal nitric oxide synthase, and tyrosine hydroxylase

**Figure 2:**
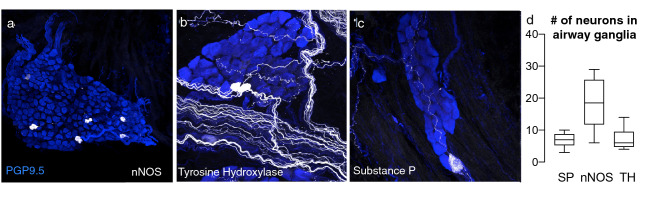
Parasympathetic nerve heterogeneity in mouse airways. Subpopulations of airway nerves express neuronal nitric oxide synthase (nNOS) (**a**), tyrosine hydroxylase (TH) (**b**), and substance P (SP) (**c**). (**d**) Quantification in WT mice: SP n = 24, nNOS n = 10, TH n = 10.

Trachea and primary bronchi from wild type C57BL/6 mice were labeled with antibodies against PGP9.5 and either SP, nNOS, or TH. Small numbers of postganglionic parasympathetic cell bodies, identified based on morphology and location within tissue, stained positively for each of these neurotransmitter markers (Fig. 2). The number of positive neurons varied between mice, with SP positive neurons representing (7 ± 2.0, n = 24 mice), nNOS (19.1 ± 6.6, n = 10), and TH (7.2 ± 3.1, n = 10) neurons per mouse (Fig. 2d).

### AAV9 transduces airway parasympathetic neurons

**Figure 3:**
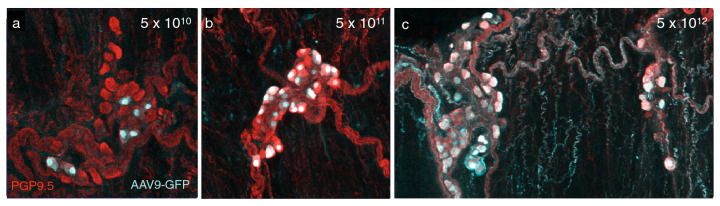
Three viral doses tested for transduction efficiency in airway neurons after intravenous injection. (**a**) Low, (**b**) medium, and (**c**) high doses tested, with successfully transduced neurons expressing GFP and all neurons labeled with PGP9.5. 5 × 10^12^ vg showed nearly 100% transduction efficiency.

We investigated the efficacy of using AAV9 viral capsids to transduce airway parasympathetic neurons. AAV9 viral capsids containing GFP driven by a CAG promoter were injected intravenously into 8 week old C57BL/6 mice at variable doses. AAV9-mediated transduction efficiency of airway parasympathetic neurons was low when a systemic dose of 5 × 10^10^ vg was used, but increased to nearly one hundred percent transduction efficiency at a dose of 5 × 10^12^ vg (Fig. 3).

### Multicolor labeling identifies individual airway parasympathetic neuronal cell bodies and their processes

**Figure 4:**
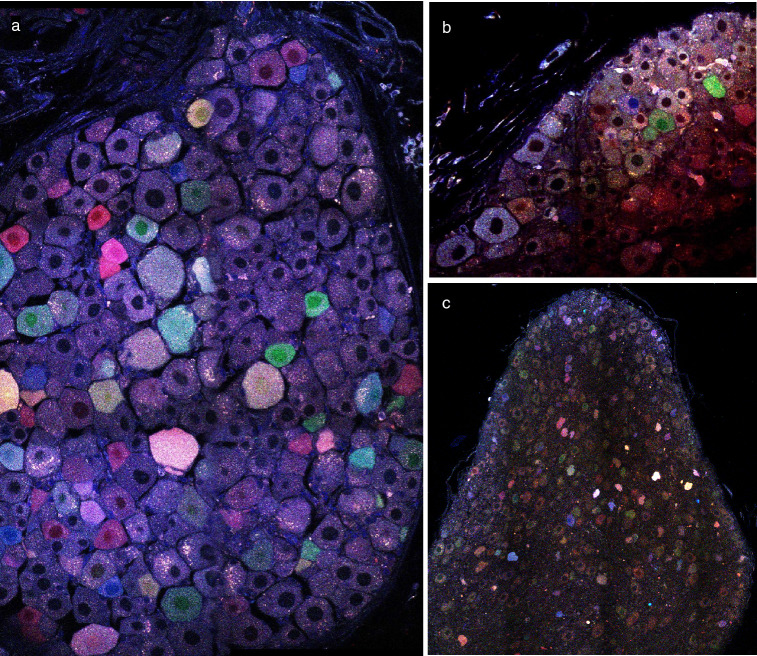
Sensory and sympathetic nerves were also transfected by multicolor AAV9. (**a**) DRG, (**b**) vagal ganglion, and (**c**) superior cervical ganglion showing nerve cell bodies labeled with multicolored AAVs. Total viral dose 1 × 10^12^.

**Figure 5:**
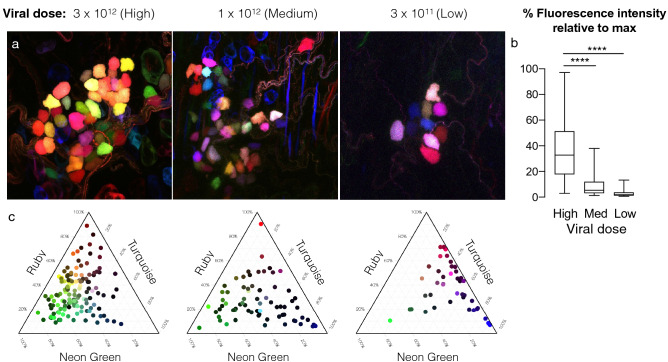
Fluorescence intensity of parasympathetic neurons dependent on viral dose. (**a**) Representative images of ganglia from three viral doses. (**b**) Fluorescence intensity represents the average intensity in a single nerve soma with the three color channels added together. Doses compared using a Kruskal–Wallis test with Dunn’s post-hoc test. *p < 0.01, ****p < 0.0001. n = 41–139. (**c**) Ternary plots for each dose were used to assess color spread, with the normalized fluorescent intensity of each neuron cell body plotted as a proportion of the three possible colors. Color variation was smaller (points clustering towards the center of the chart) in the high dose, was skewed toward the Turquoise vector in the low dose, and was most evenly distributed in the middle dose.

**Figure 6:**
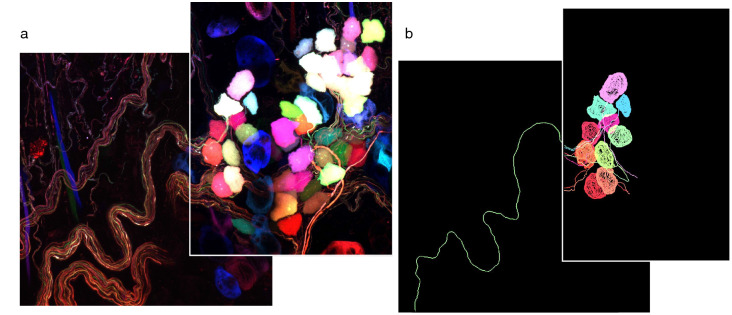
Nerves were traced using nTracer plugin for FIJI. (**a**) Original overlapping images of parasympathetic neurons. (**b**) Traces of select neurons and axons.

Three AAV plasmids from Addgene expressing the fluorescent proteins mNeonGreen, mRuby2, and mTurquoise2 were packaged into AAV9 viral capsids and injected intravenously into mice. Tissues were passively cleared by immersion in Ce3D solution to preserve endogenous fluorescence. Imaging revealed multicolor labeling of parasympathetic neurons, as identified by anatomical location (Fig. 1e). Sensory and sympathetic neurons also showed vector uptake, although these populations were not used for further tracing studies (Fig. 4). Increased viral doses increased the range of fluorescence colors as well as the intensity, although a total dose of 1 × 10^12^ vg showed the greatest color spread (Fig. 5). We used the nTracer plugin for FIJI to semi-manually trace axons (Fig. 6).

### Substance P, nNOS, and tyrosine hydroxylase do not represent intra-ganglionic interneurons

**Figure 7:**
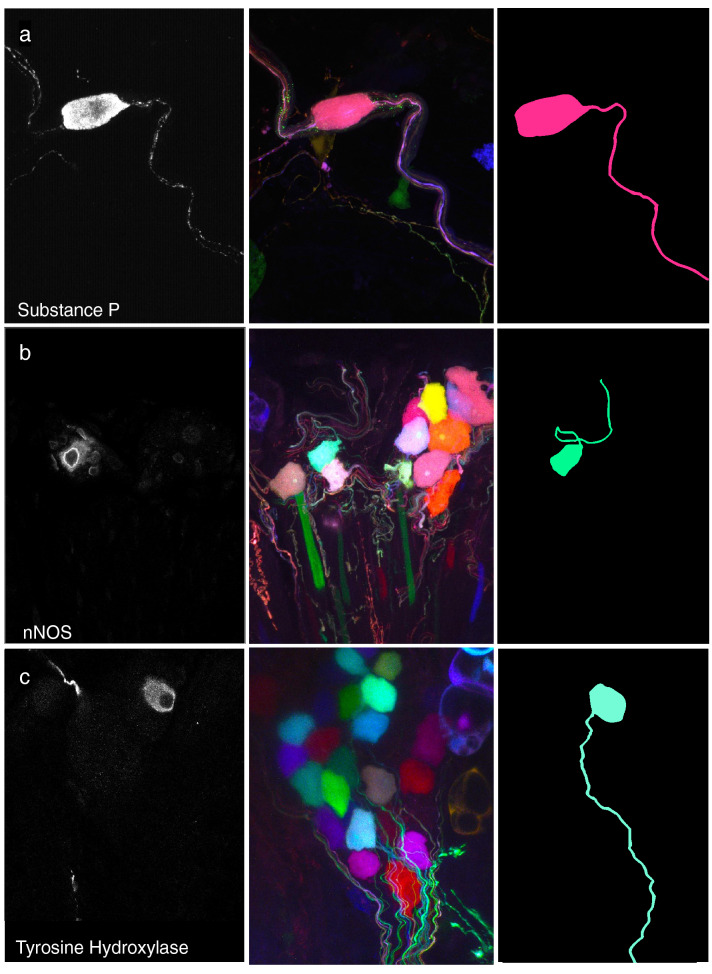
Tracheas immunolabeled for substance P (**a**), neuronal nitric oxide synthase (**b**), and tyrosine hydroxylase (**c**). Left column shows immunolabeling in a single plane. Middle column shows multicolor labeling of neurons in a maximum intensity projection. Right column shows traces of cell bodies and axons of labeled neurons. Two axons of very similar color are present in the substance P image, however only one originates from the cell body that is shown, as can be seen more clearly in a 3D/orthogonal view (see supplementary Fig. 2).

**Figure 8:**
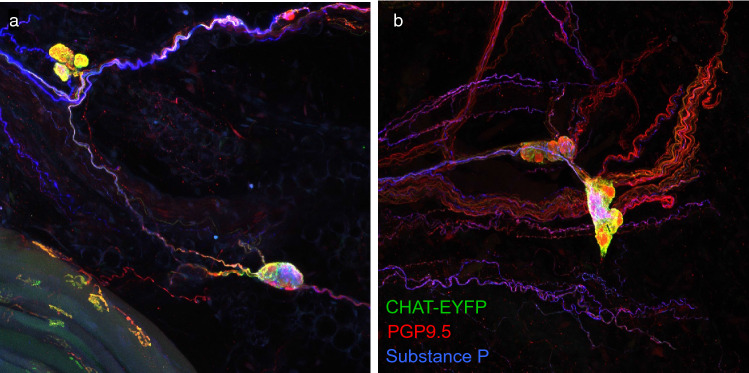
Examples of substance P (blue) nerves in parasympathetic ganglia in mice with CHAT driving expression of EYFP (green) co-stained with PGP9.5 (red). (**a**) A parasympathetic ganglion with three cell bodies and a single substance P neuron both expressing EYFP. (**b**) A densely innervated parasympathetic ganglion with one of its neurons expressing substance P and all expressing EYFP.

We co-stained AAV9-labeled tracheas with antibodies against SP, nNOS, or TH using secondary antibodies in the far-red spectrum. We identified a single SP-positive parasympathetic neuron (Fig. 7a), plus two neurons labeled with nNOS, and two with TH. The SP-positive neuron was not part of a ganglion. Neurites of nNOS and TH-positive nerves exited their respective ganglia, rather than terminating within the ganglia as interneurons (Fig. 7b,c). Co-staining for substance P in mice with GFP driven by the choline acetyltransferase (CHAT) promoter was used in addition to anatomical location around the airways to confirm the parasympathetic nature of these neurons (Fig. 8).

## Discussion

This study is the first demonstration of nNOS- and TH-expressing neurons in airway parasympathetic ganglia of mouse airways, and confirms reports of mouse parasympathetic SP expression. Our technique has exciting potential for studying the role of these neurons in airway disease.

Increased substance P in parasympathetic neurons, with pro-inflammatory and neurotransmitter roles, may have a role in airway hyperreactivity^6^. Nitric oxide is a bronchodilator, usually produced by a population of neurons with cell bodies located in the esophagus^15^. Tyrosine hydroxylase produces dopamine, epinephrine, and norepinephrine, and is usually found in sympathetic nerves, which can suppress airway parasympathetic nerves, and inhibit bronchoconstriction. While one possibility for the physiologic effects of these neurons is that they function as interneurons within ganglia, our studies show that processes from these neurons predominantly project outside their ganglia of origin to innervate distant targets.

Previous studies have seen parasympathetic neurons synapsing onto one another between ganglia^16^, but did not identify whether those nerves expressed neurotransmitters other than acetylcholine. While none of the SP, TH or nNOS neurons we identified projected to other parasympathetic neurons within their own ganglia, projection to parasympathetic neurons in other ganglia cannot be ruled out. Other neuropeptides, such as VIP and NPY, have also been identified in rodent and human parasympathetic ganglia^2,4,17^, and would be interesting to investigate with this method in future studies.

Our results indicate, for the first time, that AAV9 is capable of driving expression of fluorescent proteins in airway nerves after intravenous injection. Multicolor labeling of airway neurons using viral vectors represents a significant step forward in airway innervation research, especially given its versatility and applicability in larger animal models.

## Supplementary Information

Supplementary Figures.

## References

[1] Dey RD, Hoffpauir J, Said SI (1988). Co-localization of vasoactive intestinal peptide- and substance P-containing nerves in cat bronchi. Neuroscience.

[2] Dey RD (1996). Neurochemical characterization of intrinsic neurons in ferret tracheal plexus. Am. J. Respir. Cell Mol. Biol..

[3] Scott GD, Blum ED, Fryer AD, Jacoby DB (2014). Tissue optical clearing, three-dimensional imaging, and computer morphometry in whole mouse lungs and human airways. Am. J. Respir. Cell Mol. Biol..

[4] Fischer A, McGregor GP, Saria A, Philippin B, Kummer W (1996). Induction of tachykinin gene and peptide expression in guinea pig nodose primary afferent neurons by allergic airway inflammation. J. Clin. Investig..

[5] Springall DR (1990). Persistence of intrinsic neurones and possible phenotypic changes after extrinsic denervation of human respiratory tract by heart-lung transplantation. Am. Rev. Respir. Dis..

[6] Wu Z-X, Satterfield BE, Dey RD (2003). Substance P released from intrinsic airway neurons contributes to ozone-enhanced airway hyperresponsiveness in ferret trachea. J. Appl. Physiol..

[7] Livet J (2007). Transgenic strategies for combinatorial expression of fluorescent proteins in the nervous system. Nature.

[8] Tsuriel S, Gudes S, Draft RW, Binshtok AM, Lichtman JW (2015). Multispectral labeling technique to map many neighboring axonal projections in the same tissue. Nat. Methods.

[9] Chan KY (2017). Engineered AAVs for efficient noninvasive gene delivery to the central and peripheral nervous systems. Nat. Neurosci..

[10] Earley LF (2017). Adeno-associated virus (AAV) assembly-activating protein is not an essential requirement for capsid assembly of AAV serotypes 4, 5, and 11. J. Virol..

[11] Powers JM, Chang XL, Song Z, Nakai H (2018). A quantitative dot blot assay for AAV titration and its use for functional assessment of the adeno-associated virus assembly-activating proteins. J. Vis. Exp..

[12] Li W, Germain RN, Gerner MY (2017). Multiplex, quantitative cellular analysis in large tissue volumes with clearing-enhanced 3D microscopy (Ce3D). Proc. Natl. Acad. Sci. USA..

[13] Roossien DH (2019). Multispectral tracing in densely labeled mouse brain with nTracer. Bioinformatics.

[14] du Sert NP (2020). The ARRIVE guidelines 2.0: Updated guidelines for reporting animal research. PLoS Biol..

[15] Canning BJ, Undem BJ (1993). Relaxant innervation of the guinea-pig trachealis: Demonstration of capsaicin-sensitive and -insensitive vagal pathways. J. Physiol..

[16] Coburn RF, Kalia MP (1986). Morphological features of spiking and nonspiking cells in the paratracheal ganglion of the ferret. J. Comp. Neurol..

[17] Richardson RJ, Grkovic I, Anderson CR (2003). Immunohistochemical analysis of intracardiac ganglia of the rat heart. Cell Tissue Res..

