# Supplementary material for: Multicolor labeling of airway neurons and analysis of parasympathetic heterogeneity
Source: Sci Rep. 2022 Mar 23;12:5006. doi: 10.1038/s41598-022-08655-6 (PMC8943012; doi:10.1038/s41598-022-08655-6)
Supplement: Supplementary file 1 — Supplementary Figures. [file 41598_2022_8655_MOESM1_ESM.pdf]

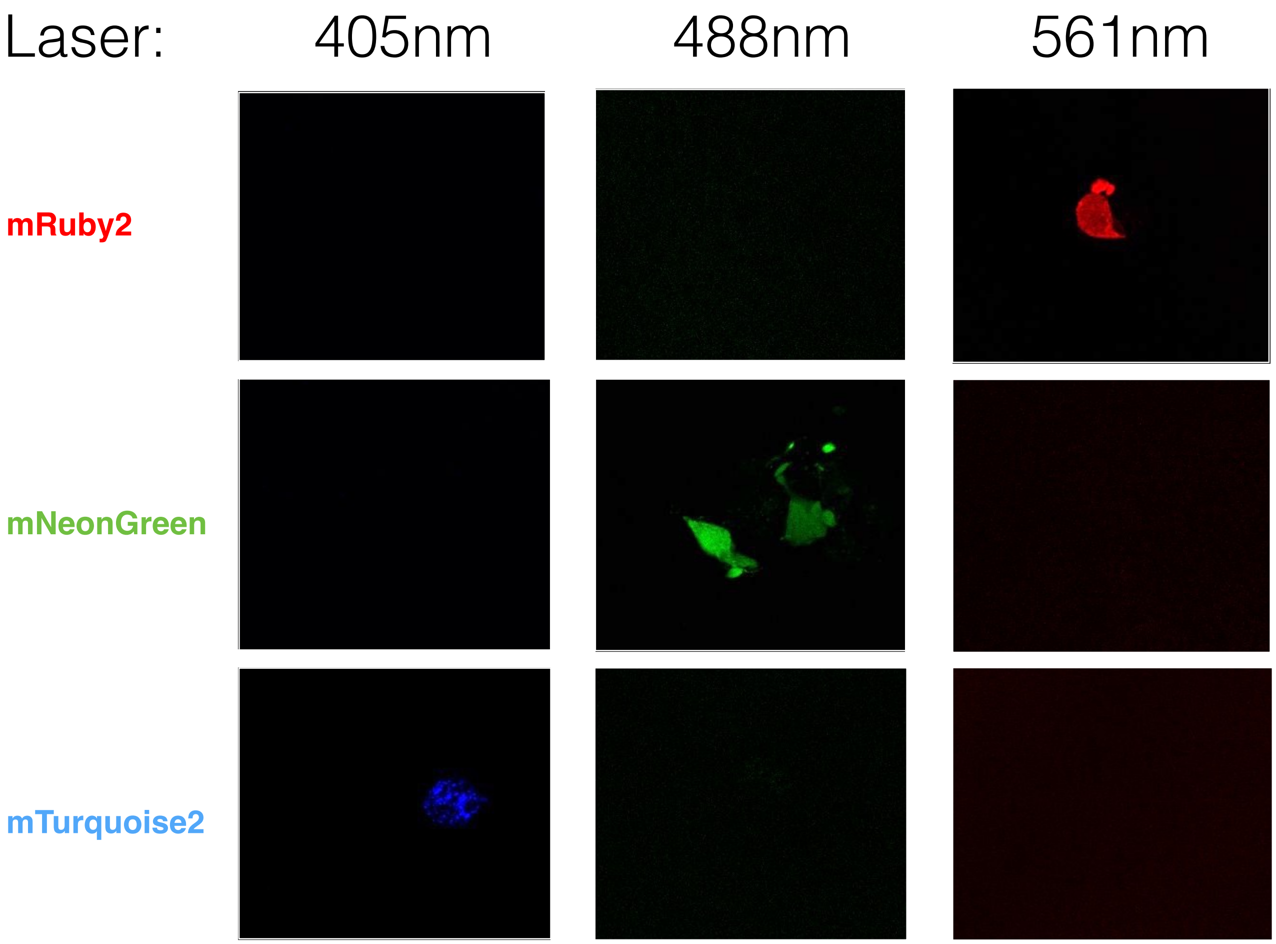

Supplementary Figure 1. Validated microscope settings in HEK293 cells. Groups of cells in separate wells were transfected with plasmids for each of the three fluorophores. Windows for detection of emitted light were narrowed such that each channel only detected light from a single fluorophore. Final microscope settings were laser 405 nm (detection wavelengths 467-503 nm), 488 nm (516-526 nm), and 561 nm (576-619 nm).

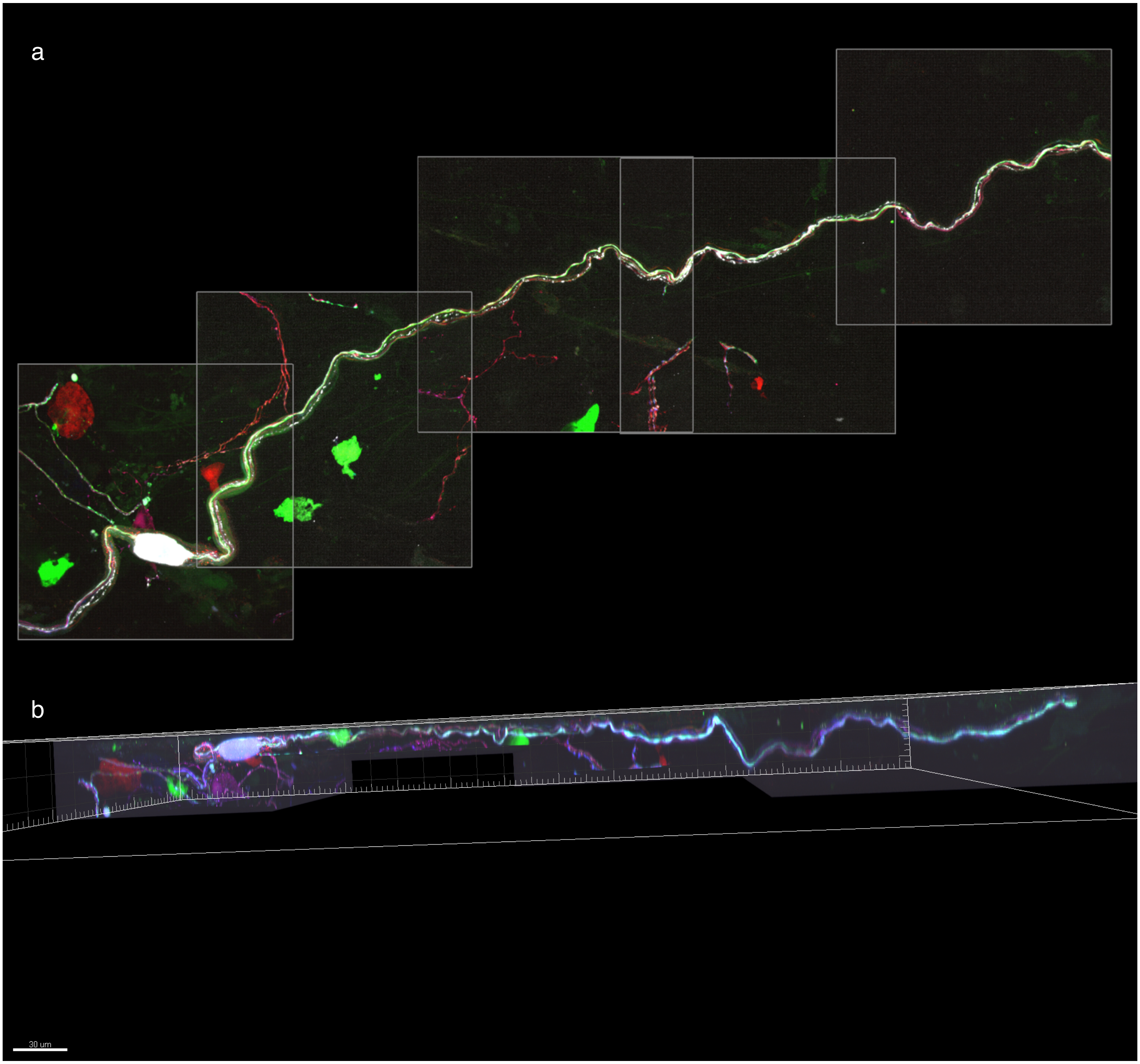

Supplementary Figure 2. Nerve labeled with multicolors and with antibody against substance P (white). (a) Many overlapping images were stitched together to trace the axon through the tissue. Image created using Imaris Stitcher. (b) Orthogonal view of substance P positive multicolor neuron shown in part a. Image created using Imaris software.
